# In vitro infection of bovine erythrocytes with *Theileria annulata* merozoites as a key step in completing the *T. annulata* life cycle in vitro

**DOI:** 10.1038/s41598-024-54327-y

**Published:** 2024-02-13

**Authors:** Khawla Elati, Shahin Tajeri, Robert M. Mugo, Isaiah Obara, Mohamed Aziz Darghouth, Erich Zweygarth, Ard Menzo Nijhof

**Affiliations:** 1 Institute of Parasitology and Tropical Veterinary Medicine, Freie Universität Berlin, Robert-Von-Ostertag-Str. 7, 14163 Berlin, Germany; 2 Veterinary Centre for Resistance Research, Freie Universität Berlin, Robert-Von-Ostertag-Str. 8, 14163 Berlin, Germany; 3 Laboratoire de Parasitologie, École Nationale de Médecine Vétérinaire de Sidi Thabet, Institution de la Recherche et de l’Enseignement Supérieur Agricoles, Université de la Manouba, 2020 Sidi Thabet, Tunisia; 4 Institute of Immunology, Center for Infection Medicine, Freie Universtät Berlin, 14163 Berlin, Germany; 5 Department of Veterinary Tropical Diseases, Faculty of Veterinary Science, University of Pretoria, Pretoria, South Africa

**Keywords:** *Theileria annulata*, In vitro infection, Merozoites, Bovine erythrocytes, Imaging, Microscopy, Cell growth, Parasite biology

## Abstract

*Theileria annulata* is a protozoan parasite with a complex life cycle involving a bovine host and a tick vector. It is transmitted by *Hyalomma* ticks and is the causative agent of tropical theileriosis, a debilitating and often fatal disease in southern Europe, northern Africa and large parts of Asia. Understanding the biology of different life cycle stages is critical for the control of tropical theileriosis and requires the use of experimental animals which poses an ethical concern. We present for the first time the in vitro infection of red blood cells (RBCs) with *T. annulata* differentiated schizonts. The Ankara cell line of *T. annulata* was cultured at 41 °C for nine days to induce merogony and subsequently incubated with purified RBCs for one to three days. Percentage of parasitized erythrocyte (PPE) over the short culture period was estimated by Giemsa staining (0.007–0.01%), Flow cytometry activated sorting (FACS) (0.02–1.1%) and observation of FACS sorted cells by confocal microscopy (0.05–0.4%). There was a significant difference in the PPE between FACS and the two other techniques (one-way ANOVA followed by Tukey test, P = 0.004) but no significant difference was observed between the confocal imaging and Giemsa staining methods (ANOVA one-way followed by Tukey test, P = 0.06). Importantly, all three complementary methods confirmed the invasion of RBCs by *T. annulata* merozoites in vitro. Although the experimental conditions will require further optimization to increase the PPE, the in vitro infection of RBCs by *T. annulata* merozoites is pivotal in paving the way for the eventual completion of the *T. annulata* life cycle in vitro when combined with artificial tick feeding.

## Introduction

Cattle theilerioses, including diseases caused by *Theileria annulata* and *Theileria parva*, are tick-transmitted and cause serious impediments to livestock productivity in Africa, Southern Europe and Asia, resulting in a large economic burden. Tropical theileriosis caused by *T. annulata* occurs in Northern Africa, Southern Europe and large part of Asia where *Hyalomma* vector ticks occur^1^, while East Coast fever, caused by *T. parva*, is transmitted by *Rhipicephalus appendiculatus* ticks distributed in large parts of eastern, central and southern Africa^2–4^. For *T. parva*, the losses were estimated to be 300 million US$, with a mortality of 1.1 million cattle annually only in sub-Saharan Africa^5,6^. For *T. annulata*, the total losses were for instance estimated to reach 598,133 US $ for 2 years in endemic regions in Turkey only^7^.

Both protozoan parasites have similar life cycles involving a mammalian host and a tick vector. For *T. annulata*, *Hyalomma* ticks infect cattle by releasing sporozoites with their saliva during feeding on bovine hosts. The sporozoites infect leukocytes and after differentiation to macroschizonts induce uncontrolled proliferation of the infected cell, resulting in a cancer-like phenotype. The macroschizonts undergo merogony and produce a large number of microschizonts and merozoites, which upon rupture of the infected cell are released into the circulation and actively invade erythrocytes where they develop further into piroplasms. Ticks become infected with *Theileria* when they imbibe infected erythrocytes during a blood meal^8,9^.

Studies involving different life stages of *T. annulata* (schizonts, piroplasms and sporozoites) and parasite-vector interactions often require the use of experimental animals, which is of ethical concern. Artificial tick feeding systems (ATFSs) based on silicone membranes or animal-derived membranes have been successfully adapted for the tick vectors of *T. annulata*, such as *Hyalomma dromedarii, H. anatolicum*^10^, *H. lusitanicum*^11^, *H. excavatum* and *H. marginatum*^12^. *Theileria* transmission has also been studied in vitro using naturally infected blood. *Hyalomma anatolicum* nymphs were for instance naturally infected with *Theileria lestoquardi-*infected blood in vitro using an ATFS^13^ and *Rhipicephalus appendiculatus* adults that were infected with *T. parva* as nymphs by acquisition feeding on an infected calf were demonstrated to secrete infective *T. parva* sporozoites with their saliva into an ATFS^14^. Both examples depended on the use of experimental animals to obtain the piroplasm stage infective to ticks.

There has been only one reported attempt to infect erythrocytes in vitro with *T. annulata* schizonts^15^. In that experiment, assessment of invasion was solely based on the examination of Giemsa-stained blood smears, which lacks sensitivity as piroplasms cannot always be clearly distinguished from artefacts to confirm successful invasion. Additional methods to confirm invasion are therefore required, as for instance established for *Plasmodium falciparum*, where successful invasion of RBCs with *P. falciparum *in vitro has been validated using additional cytoplasmic and nuclear dyes^16–18^.

In the present work, we confirmed infection of RBCs with *T. annulata* merozoites generated in vitro using a combination of flow cytometry and confocal microscopy, in addition to the classical Giemsa staining method. We recently demonstrated that it is possible to successfully feed all stages of the *T. annulata* vectors *H. dromedarii*, *H. excavatum* and *H. scupense *in vitro on silicone-based membranes (Elati et al*.*, 2023, submitted). If this could be combined with the feeding of RBCs infected with *T. annulata* merozoites in vitro, it would bring the objective of completing the full lifecycle of *T. annulata *in vitro one step closer*.*

## Results

**Figure 1:**
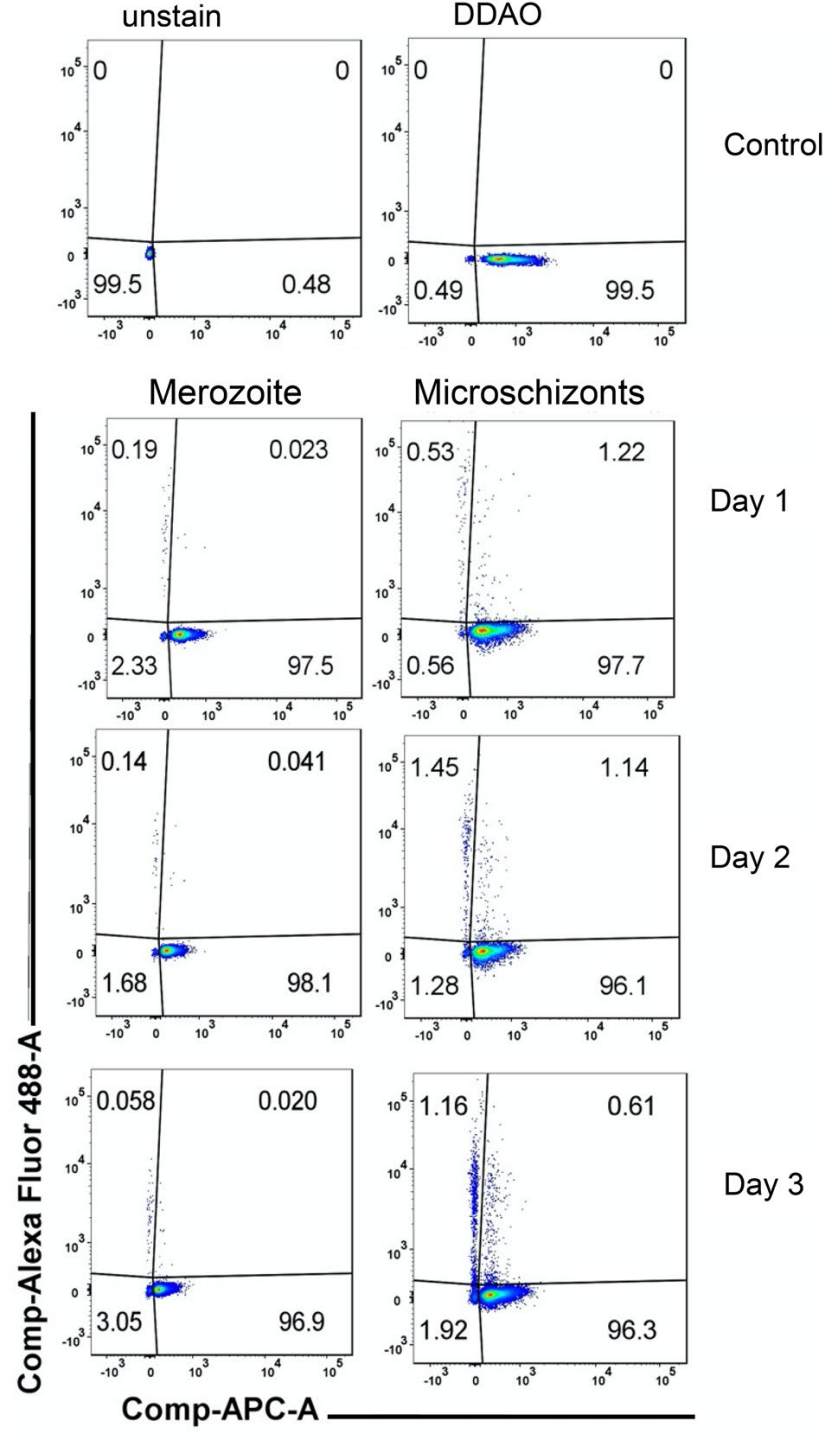
Infection of RBCs by *T. annulata*, flow-cytometry dot-plots (APC-DDA versus Alexa Fluor 488A-SYBR Green) showing the percentage of infected erythrocytes (PPE) incubated with *T. annulata* merozoites (left) or microschizont-infected leukocytes (right) for one, two or three days of cultivation. Unstained cells and RBCs stained only with DDAO (single stain) served as control.

The infection of RBCs with *T. annulata* merozoites or schizonts was confirmed by three techniques that showed different percentages of infection. Flow cytometry results with SYBR green I and DDAO staining showed that RBCs incubated with *T. annulata* schizonts-infected leukocytes have higher PPE than cells incubated with merozoites. The microschizonts-infected PPE decreased from 1.22% at day 1 to 0.61% at day 3 (chi-square test, P < 0.0001). While the cells infected with merozoites had a lower infection rate from the first to the third day, ranging from 0.02 to 0.04% (chi-square test, P = 0.1) (Fig. 1).

**Figure 2:**
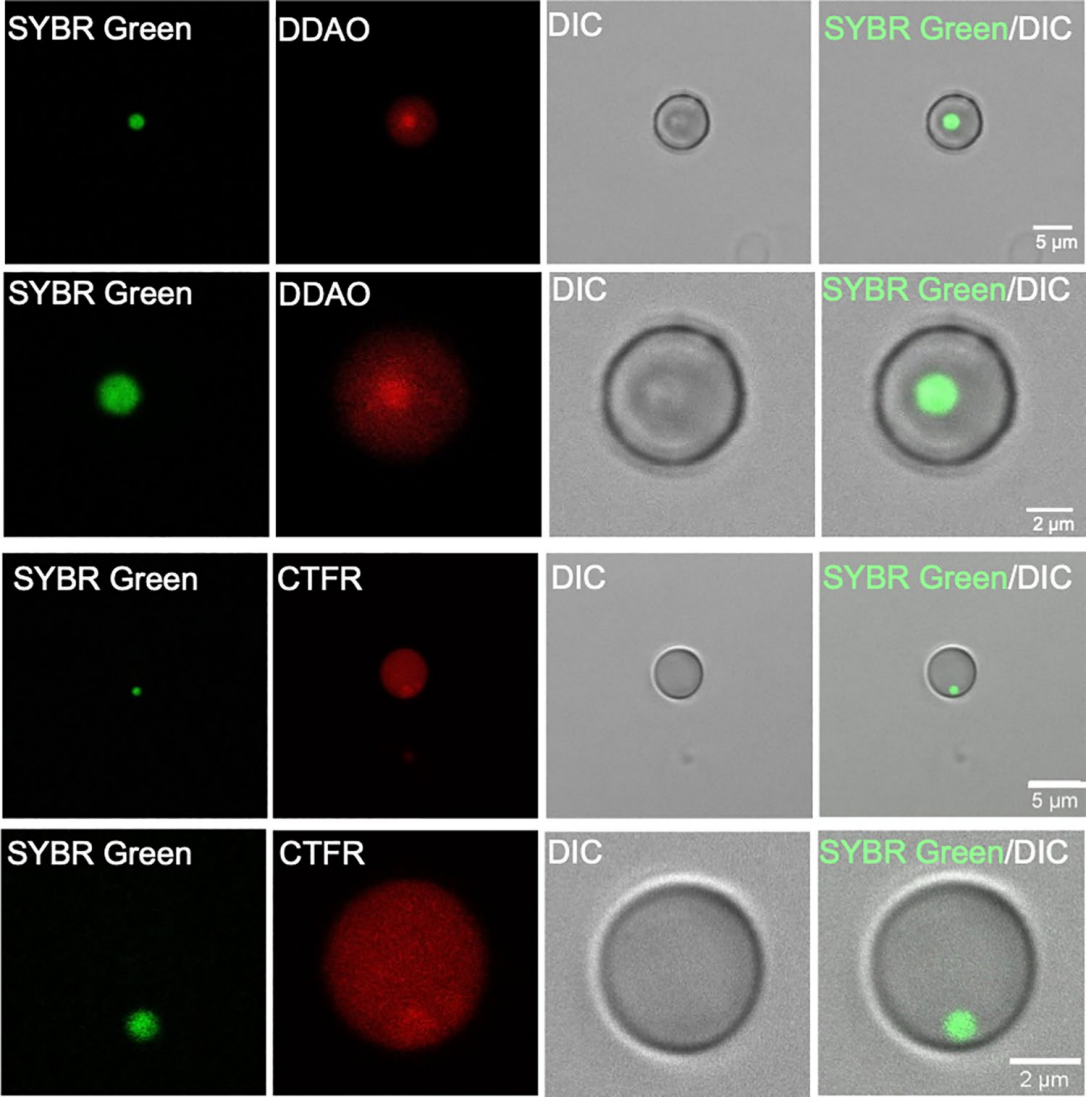
Bovine red blood cells infected with *T. annulata *in vitro and observed with confocal microscopy using DDAO or CTFR (red) for staining of the erythrocytes and SYBR green I (green) for DNA staining of piroplasms. *DIC* differential interference contrast microscopy.

The sorted cells observed by confocal microscopy showed clear infection of RBCs with *T. annulata* piroplasms (Fig. 2) but with lower infection rates compared to FACS. Free parasites and uninfected RBCs were also observed. The PPE varied between 0.05% and 0.4% for cells incubated with microschizonts-infected leukocytes (Chi-square test, P = 0.01) while no infected cells were observed for RBCs incubated with merozoites (Fig. 4).

**Figure 3:**
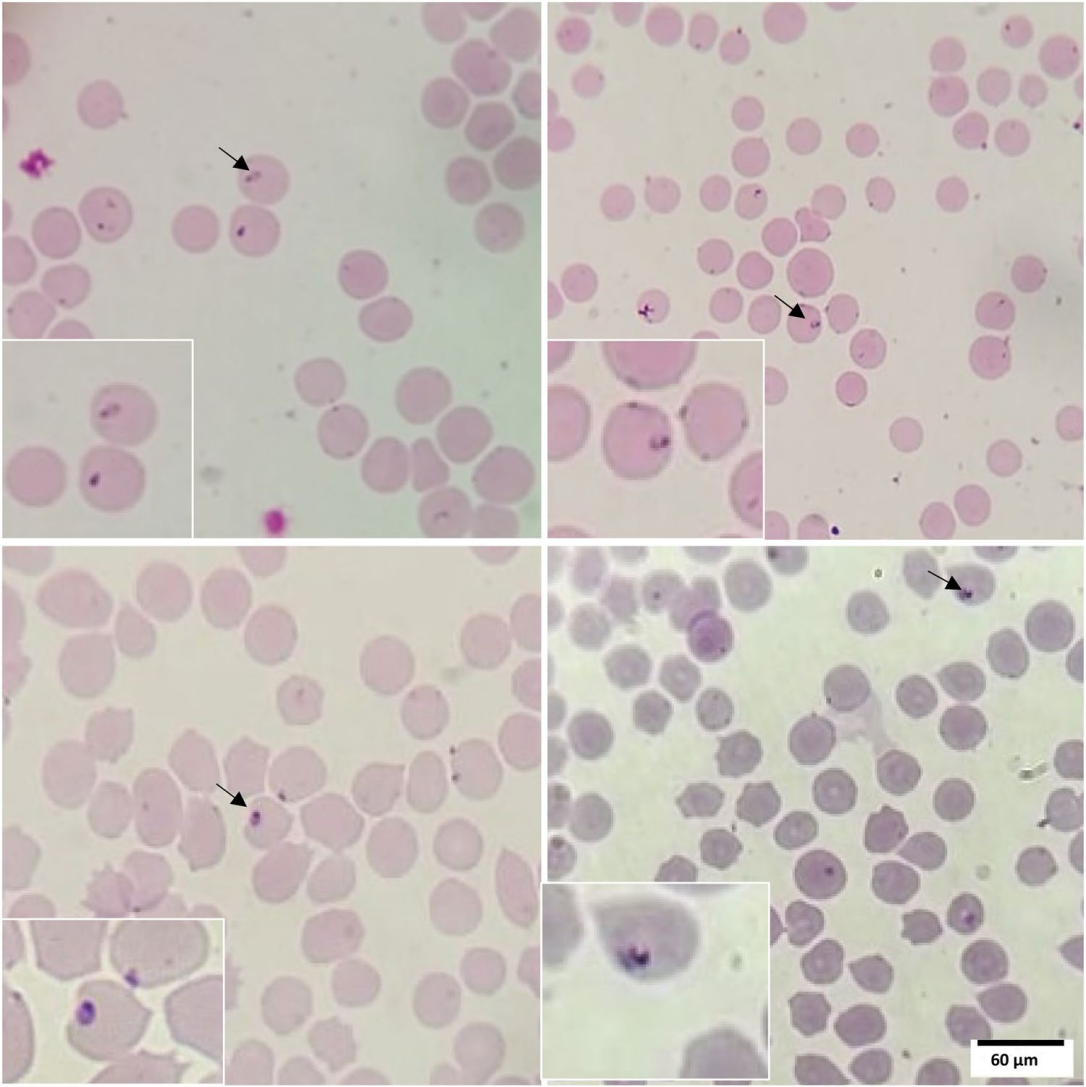
Giemsa-stained blood smear prepared from in vitro co-cultures of bovine RBCs and *T. annulata* merozoites/microschizonts. The piroplasm-like forms of the parasite are clearly visible within the infected RBCs.

**Figure 4:**
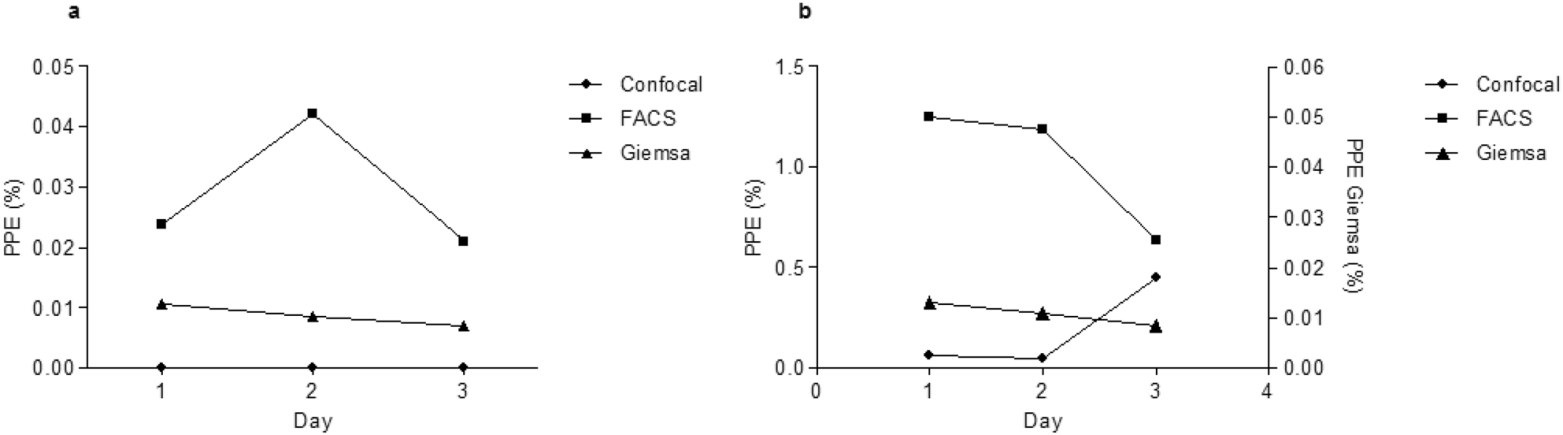
Variation of percentage of parasitized erythrocytes (PPE) incubated with merozoites (**a**) or with microschizont-infected leukocytes, (**b**) over 3 days of culture.

The microscopic observation of Giemsa-stained thin smears from the same culture on different days confirm the infection of RBCs with *T. annulata* piroplasms (Fig. 3) but the PPE also varied between 0.007 and 0.01% for merozoite-infected cells with (Chi-square test, P = 1) and between 0.008 and 0.01% for cells infected with microschizonts (Chi-square test, P = 0.5), lower than the value observed with FACS and confocal microscopy (Fig. 4).

The PPE varied significantly between FACS and both confocal microscopy and Giemsa staining techniques for cells incubated with merozoites or microschizonts-infected leukocytes (ANOVA one-way followed by Tukey test, P = 0.004). While no significant difference was observed between confocal imaging and Giemsa staining (ANOVA one-way followed by Tukey test, P = 0.06).

## Discussion

The present work aimed to infect RBCs with *T. annulata *in vitro, and evaluate the invasion using three complimentary techniques, without the need for experimental animals.

In this study, we followed four key steps to infect and validate the invasion of erythrocytes with *T. annulata *in vitro*.* These were: (i) induction of merogony from an initially established schizont-transformed leukocyte culture, (ii) collection of blood samples and isolation of erythrocytes, (iii) infection of erythrocytes by mixing of the differentiated *T. annulata* parasites with the isolated RBCs and subsequent incubation at 37 °C and (iv) validation of invasion using three complementary methods allowing for a calculation of the PPE over time.

Our choice of techniques for confirming invasion of erythrocytes was informed by findings from previous experiments assessing invasion of erythrocytes by *Plasmodium* spp. by combining different methods such as Giemsa staining, immunofluorescence microscopy, flow cytometry, PCR-based methods and confocal microscopic imaging^17,19,20^. It is important to emphasise that a combination of methods was shown to be essential for confirmation of pathogen invasion into erythrocytes^20^.

Giemsa staining, FACS and confocal microscopy all confirmed the invasion of *T. annulata* merozoites into RBCs over a short culture period, albeit with low parasitaemias. The lowest PPE was recorded for the Giemsa-stained samples (0.007–0.01%) followed by confocal microscopy (0.05–0.4%). The highest PPE was recorded by FACS (1.2%) for cells incubated with schizont-transformed leukocytes on the first day of culture. FACS is a sensitive and powerful technique that allows for the detection of low parasitaemia levels, which is particularly helpful when the number of infected erythrocytes is low. The sensitivity of FACS depends on the fluorescent labels used and the sensitivity of the flow cytometer^20,21^. One possible explanation for the relatively high PPE estimated by FACS is that it potentially includes the parasites present on the surface of the cells, which are cleaved during cell sorting and this may explain both uninfected RBCs and free parasites were observed by confocal microscopy after sorting.

There are some limitations that must be taken into account when using FACS for this purpose. The first factor to consider is sample preparation. FACS requires that the cells are in a single-cell suspension for analysis. Achieving a single-cell suspension from whole blood samples can be challenging especially when erythrocytes are infected with the parasite. The presence of cell debris, clumped cells, or host cell aggregates may interfere with accurate analysis and lead to incorrect assessment of parasitaemia. Moreover, loading a large amount of sample requires a lot of time for analysis, which is why only ten microliters of culture were used for FACS sorting in this study.

In addition, flow cytometry instruments and reagents are expensive, specialized training is required to operate the equipment and accurate analysis of the data and the whole process is time-consuming.

Despite these limitations, flow cytometry was shown to be a valuable tool for assessing infected erythrocytes. Combining flow cytometry with other complementary techniques can improve accuracy and provide more comprehensive information about the dynamics of parasite infection in erythrocytes.

Giemsa staining is usually a technique used in acute phases of infection but has limited use during the carrier state of infection when low parasitaemia are present, due to the risk of obtaining false negative results or underestimating parasitaemia^22^. As for the confocal microscopy, although we saw clear evidence for the infection of RBCs with *T. annulata,* we were unable to fix the sorted cells on glass slides, mainly due to photobleaching. Fixing the cells and improving the quality of the captured images will be a priority for future research.

It is possible that the low parasitaemia observed in this study could be attributed to the type of medium and serum used. For example, it has previously been shown for *Theileria uilenbergi* that the use of HL-1 medium supported the growth of the intraerythrocytic parasite for longer periods in culture^23^. Other studies showed that equine piroplasms grew better in M199 supplemented with 40% FBS where the heat inactivation of the serum negatively affected the growth and survival of the culture, thus suggesting an essential role of the complement system in the continuous culture of erythrocytic stages of *Theileria equi* (formerly referred to as *Babesia equi*)^24^.

The complement system was also required for successful invasion of RBCs by *Babesia rodhaini*^25^. The addition of other supplement such as bovine serum albumin (BSA), lipids-cholesterol-rich mixture, insulin, transferrin, selenite, and putrescine could help improve the PPE and have been shown to replace the use of FBS in *Plasmodium* and *Babesia* cultures^26,27^. The purine compounds are also crucial for the growth of the parasite due to their inability to synthesise it, which justifies the use of hypoxanthine^23,24^.

To improve schizont to merozoite differentiation yield, other methods based on chemical compounds such as apicidin^28^ and colchicine^29^ could be used. It was also reported that merogony could be induced using apicomplexan-specific histone deacetylase inhibitor FR235222, similar to Apicidin, which could be combined with elevated temperatures (41 °C) suggesting that two merogony inducing stimuli might act synergistically to generate higher merozoite numbers or more infectious merozoites^30^.

Infection of RBCs with *Theileria* has been attempted for six strains of *T. parva* using microschizonts and free merozoites incubated at 37 °C in normal atmosphere (5% CO_2_) and RMPI supplemented with 20% FBS but no penetration of merozoites to RBCs was observed in Giemsa-stained smears^31^. A single study showed the possible infection of bovine, ovine and caprine erythrocytes in vitro with *T. annulata* macroschizonts also used Giemsa staining, where the first piroplasm form was observed at day 19 for bovine RBCs, with a parasitaemia level varying from 0.01% to 0.03%^15^. There are several ways in which the present findings extend previous research. Foremost, we use a combination of three complementary methods to confirm the invasion of RBCs by *T. annulata* merozoites in vitro. As mentioned, this is an important step towards completion of the life cycle in vitro as techniques like artificial tick feeding can be utilized to feed infected erythrocytes to tick vector. The other key methodological improvement in the generation of infected erythrocytes relates to the duration of incubation before successful infection of erythrocytes. Herein, piroplasms were observed from the first day of culture as opposed to the 19 days previously reported^15^. This is likely attributable to the fact that we incubated the RBCs with cells already differentiated into microschizonts and free merozoites. Also, important to note is that we used different culture conditions (reduced oxygen levels) while the previous two studies^15,31^ used normal culture conditions with CO_2_. One other important difference was in the composition of the media, as we used hypoxanthine which is necessary for the growth of the parasite.

Although several studies have been conducted on the mechanism used by *T. annulata* sporozoite to invade and transform bovine leukocytes^32,33^, the process of invasion of RBCs by *T. annulata* merozoites has not yet been investigated. For the closely related *T. parva*, it has been demonstrated that the mechanism of entry of merozoites into RBCs is similar to that of sporozoite invasion^32,34^. There are far more studies on *Plasmodium* invasion of RBCs and their in vitro culture which is facilitated by the fact that re-invasion of the parasite into new RBCs takes only 1–2 min^35^. Furthermore, the structure of infected RBCs is altered to improve their survival resulting in greater rigidity, adhesion and permeability of the cell membrane^36^. The validated infection of RBCs in vitro and the establishment of a continuous culture of the piroplasm stage of *T. annulata* will serve as starting point for conducting further studies to understand the invasion process of *T. annulata* merozoites.

We present for the first time a validation of infection of bovine RBCs with *T. annulata* merozoites in vitro and confirmed the invasion using three complimentary techniques. The current findings set the stage for additional experiments aimed at increasing the number of parasitized RBCs and maintaining a continuous culture of the intra-erythrocytic stages of the parasite. Ultimately, these infected erythrocytes will be an important material for the infection of ticks thus completing the life cycle of *T. annulata *in vitro while at the same time adhering to the 3Rs principle in humane animal research by reducing the use of animals for this purpose. This model could also be applied to other pathogens such as *T. parva* and *T. lestoquardi* which have similar life cycles. Studying in vitro infection of erythrocytes with *T. annulata* can help researchers to understand not only the molecular mechanisms that control the interaction between the parasite and host cells but also the invasion process.

## Materials and methods

### Parasite culture and purification of microschizonts and merozoites

The *T. annulata* Ankara cell line at passage 4 established as previously described^37^ was propagated in a 75 cm^2^ culture flask containing 50 ml RPMI 1640 medium supplemented with 10%(v/v) heat inactivated foetal bovine serum (FBS). The medium was buffered with 20 mM HEPES (*N*[2-hydroxyethyl] piperazine-*N*′-[2-ethanesulfonic acid]) and supplemented with 2 mM l-alanyl-lglutamine, 100 IU/ml penicillin and 100 μg/ml streptomycin. The culture was placed at 41 °C for nine days in order to induce merogony and obtain a sufficient number of merozoites^38^. The culture was diluted (1:1) at day 2. At day 9, merozoites were isolated by differential centrifugation^38^ from an initial number of schizonts estimated to be 1.45 × 10^6^ cells/ml. The cells were first centrifuged at 120*g* for 5 min, resulting in a pellet that mainly contained undifferentiated cells infected with macroschizonts. The supernatant was then centrifuged at 1000*g* for 15 min, the pellet that was formed during this step mainly contained microschizonts. A third spin of the supernatant at 4300*g* for 15 min was performed to obtain the merozoites-enriched pellet. The pellets from the second and the third centrifugation step containing microschizonts and free merozoites were separately used to infect RBCs as described below.

### Non-parasitized erythrocytes for initiation of cultures

Blood was collected in tubes containing heparin as an anticoagulant from a piroplasm-free bovine calf and centrifuged at 800×*g* for 10 min. Plasma and buffy coat were discarded and the cell pellet containing the RBCs was washed 3–4 times with sterile modified Vega Y Martinez solution (mVYM)^24^ or 10 ml of phosphate-buffered saline (PBS) without calcium and centrifuged at 800×*g* for 10 min. After the final wash, the supernatant was removed and the cell pellet was used in initiating cultures.

### Sample preparation: parasite and red blood cells staining

Cells from the washed pellet were stained and used in initiating cultures. Staining procedures of erythrocytes with dichloro dimethyl acridin one succinimidyl ester (DDAO-SE) or Cell trace far-red (CTFR) (Thermo Fisher Scientific, United Kingdom) were adapted from previously described methods for *P. falciparum*^17,18^. The erythrocytes were stained with 20 mM DDAO-SE or 5 mM CTFR in complete RPMI medium and incubated under dark condition at 37 °C while shaking (150 r/min) for 2 h. The cells were subsequently pelleted by centrifugation at 2000 r/min (800×*g*) and incubated in RPMI complete medium for 10 min at room temperature to remove excess dyes. Finally, the pellets were washed three times at 2000 r/min (800×*g*) for 3 min with PBS. The resulting pellets were suspended in complete RPMI medium and stored at 4 °C until use. For the initial experiments both CFTR or DDAO were used, for later experiments only DDAO was used. Both stains had similar staining effectiveness when observed with confocal microscopy.

The parasite DNA was labelled using DNA-specific dye 1:1000 SYBR Green I (same sample volume) (Lonza, Rockland, ME, USA) and incubated for one hour at 37 °C in a shaking incubator. The cells were then washed once with PBS and assessment of invasion was done by flow cytometry^18^. The invasion rate was calculated as the percentage of RBCs positive for both the cytoplasmic dye DDAO and SYBR Green I.

### Initiation and maintenance of cultures

RPMI 1640 (Gibco) supplemented with 20 mM HEPES (*N*-2 hydroxymethylpiperazine *N*1-2 ethanosulfonic acid) as buffer, 20% of foetal bovine serum (FBS), with 2 mM l-alanyl-l-glutamine, 100 IU/ml penicillin and 100 μg/ml streptomycin, 500 μl l-cysteine hydrochloride (200 mM), 250 μl of bathocuproine sulfonate (8 mM) and 200 μl of hypoxanthine (100 mM) was used for the culture^23,24,39,40^.

The stained RBCs (10 μl containing approximately 15 × 10^6^ RBCs) were placed in a 96-well plate after sedimentation. Purified and labelled merozoites in 190 μl of RPMI 20% FBS medium was subsequently added to the well and carefully mixed with the RBCs. The mixture was incubated at 37 °C in a humidified 5% CO_2_, 2% O_2_, and 93% N_2_ atmosphere for 24 h, 48 h and 72 h. The medium was changed every 24 h for cells. Uninfected erythrocytes served as a negative control.

### Assessment of cell viability

The fixable viability dye eFluor 506 (eBioscience, UK) was used as an exclusion dye for dead cells. The stock solution was diluted 1:1000. For 10^7^ cells, 100 μl of dye (10 μl per 1 × 10^6^ cells in suspension) was mixed thoroughly followed by incubation for 15 min at 4 °C under protection from light^41^. Finally, the cells were washed twice with PBS and analysed using flow cytometry.

### Fluorescence-activated cell sorting of parasitised red blood cell

Stained RBCs were analysed using a BD FACSAria III (BD Biosciences, Germany) cell sorter. Total erythrocytes were gated using forward scatter area and side scatter area. RBCs singlets were then gated using the forward scatter height versus the forward scatter area followed by dead cells exclusion. Further, parasitised RBCs were identified using DDAO/CTFR (APC fluorophore) and SYBRGreen I (Alexa Fluor 488). Cells were sorted in ibidi microdishes (35 mm high) (ibidi GmbH, Gräfelfing, Germany). FlowJo software version 10 (Ashland, Becton, Dickinson and Company, 2023) was used for flow cytometry data analyses.

### Giemsa staining and microscopic examination

Thin blood smears were prepared from the culture mixture on slides on different days and were stained for 40 min in 5% Giemsa solution (Merck, Darmstadt, Germany). Due to the low number of sorted cells, fixation using methanol and staining with Giemsa was not feasible as the cells were washed off during fixation. Smears were observed under a light microscope at × 1000 magnification to detect *T. annulata-*infected RBCs in 150 fields examined*.* The percentage of parasitized erythrocytes (PPE) was calculated as follows:

$$\mathrm{PPE }\left({\%}\right)=100 \times (\frac{\mathrm{number\, of \, infected \, erythrocytes}}{\mathrm{number \, of\, examined \, erythrocytes}})$$

### Confocal microscopy and image acquisition

The microdishes with the sorted cells were viewed using immersion oil (× 63) on a Leica DMI 8 confocal microscope and images were acquired using LAS X (version 4.5.0.25531) software. Laser power was adjusted to 498 for Alexa 488 and to 638 for APC corresponding to SYBR green and DDAO, respectively. All acquired images were processed using Fiji ImageJ 2 version 2.9/1.53t^42^.

### Statistical analyses

The chi-square test for trend was used to compare the infection rate over time using an online version of Epitools (http://epitools.ausvet.com.au). A one-way ANOVA test was used to compare the difference of PPE between the three different techniques using GraphPad Prism 5 (GraphPad Software, San Diego California, United States). For both tests, a 5% threshold value was considered statistically significant.

### Ethical approval

Permission to collect bovine blood from the calf was granted by the Landesamt für Gesundheit und Soziales, Berlin, Germany (LAGeSo) under registration number H0387/17. Blood was collected by a trained veterinarian and the animal was kept in a stable at the Institute for Parasitology and Tropical Veterinary Medicine of Freie Universitaet Berlin.

## Data Availability

All data generated or analysed during this study are included in this published article.

## References

[1] Gharbi M (2020). Current status of tropical theileriosis in Northern Africa: A review of recent epidemiological investigations and implications for control. Transbound. Emerg. Dis..

[2] Bishop R, Musoke A, Morzaria S, Gardner M, Nene V (2004). *Theileria*: intracellular protozoan parasites of wild and domestic ruminants transmitted by ixodid ticks. Parasitology.

[3] Gachohi J, Skilton R, Hansen F, Ngumi P, Kitala P (2012). Epidemiology of East Coast fever (*Theileria parva* infection) in Kenya: Past, present and the future. Parasit. Vectors.

[4] Young, A. S. The epidemiology of theileriosis in East Africa. In *Advances in the Control of Theileriosis: Proceedings of an International Conference held at the International Laboratory for Research on Animal Diseases in Nairobi*, 9th–13th February 1981 (eds. Irvin, A. D., Cunningham, M. P. & Young, A. S.). 38–55 10.1007/978-94-009-8346-5_3 (Springer, 1981).

[5] Bishop RP (2020). A review of recent research on *Theileria parva*: Implications for the infection and treatment vaccination method for control of East Coast fever. Transbound. Emerg. Dis..

[6] Mukhebi AW, Perry BD, Kruska R (1992). Estimated economics of theileriosis control in Africa. Prev. Vet. Med..

[7] Inci A (2007). Economical impact of tropical theileriosis in the Cappadocia region of Turkey. Parasitol. Res..

[8] Liu J, Guan G, Yin H (2022). *Theileria annulata*. Trends Parasitol..

[9] Mehlhorn H, Shein E (1984). The piroplasms: Life cycle and sexual stages. Adv. Parasitol..

[10] Tajeri S, Razmi GR (2011). *Hyalomma anatolicum anatolicum* and *Hyalomma dromedarii* (Acari: Ixodidae) imbibe bovine blood in vitro by utilizing an artificial feeding system. Vet. Parasitol..

[11] González J, Valcárcel F, Aguilar A, Olmeda AS (2017). In vitro feeding of *Hyalomma lusitanicum* ticks on artificial membranes. Exp. Appl. Acarol..

[12] Bilgiç HB (2023). In vitro feeding of *Hyalomma excavatum* and *Hyalomma marginatum* tick species. Parasitol. Res..

[13] Tajeri S, Razmi G, Haghparast A (2016). Establishment of an artificial tick feeding system to study *Theileria lestoquardi* infection. PloS One.

[14] Vimonish R (2021). Isolation of infectious *Theileria parva* sporozoites secreted by infected *Rhipicephalus appendiculatus* ticks into an in vitro tick feeding system. Parasit. Vectors.

[15] Li Y (2014). Infection of small ruminants and their red blood cells with *Theileria annulata* schizonts. Exp. Parasitol..

[16] Theron M, Cross N, Cawkill P, Bustamante LY, Rayner JC (2018). An in vitro erythrocyte preference assay reveals that *Plasmodium falciparum* parasites prefer Type O over Type A erythrocytes. Sci. Rep..

[17] Theron M, Hesketh RL, Subramanian S, Rayner JC (2010). An adaptable two-color flow cytometric assay to quantitate the invasion of erythrocytes by *Plasmodium falciparum* parasites. Cytom. Part J. Int. Soc. Anal. Cytol..

[18] Thiam LG (2020). Feature article: Cell trace far-red is a suitable erythrocyte dye for multi-color *Plasmodium falciparum* invasion phenotyping assays. Exp. Biol. Med..

[19] Bei AK (2010). A flow cytometry-based assay for measuring invasion of red blood cells by *Plasmodium falciparum*. Am. J. Hematol..

[20] Jang JW (2014). Flow cytometric enumeration of parasitemia in cultures of *Plasmodium falciparum* stained with SYBR Green I and CD235A. Sci. World J..

[21] Vorobjev IA (2012). Optimization of flow cytometric detection and cell sorting of transgenic *Plasmodium* parasites using interchangeable optical filters. Malar. J..

[22] Mans BJ, Pienaar R, Latif AA (2015). A review of *Theileria* diagnostics and epidemiology. Int. J. Parasitol. Parasites Wildl..

[23] Miranda JPG (2006). Establishment of optimal conditions for long-term culture of erythrocytic stages of *Theileria uilenbergi*. Am. J. Vet. Res..

[24] Zweygarth E, Just MC, de Waal DT (1995). Continuous in vitro cultivation of erythrocytic stages of *Babesia equi*. Parasitol. Res..

[25] Chapman WE, Ward PA (1977). *Babesia rodhaini*: requirement of complement for penetration of human erythrocytes. Science.

[26] Ofulla AV (1993). Cultivation of *Plasmodium falciparum* parasites in a serum-free medium. Am. J. Trop. Med. Hyg..

[27] Rojas-Martínez C (2018). Babesia bigemina: Advances in continuous in vitro culture using serum-free medium supplemented with insulin, transferrin, selenite, and putrescine. Parasitol. Int..

[28] Darkin-Rattray SJ (1996). Apicidin: A novel antiprotozoal agent that inhibits parasite histone deacetylase. Proc. Natl. Acad. Sci. USA.

[29] Fritsch FM, Mehlhorn H, Schein E, Hauser M (1988). The effects of drugs on the formation of *Theileria annulata* merozoites in vitro. Parasitol. Res..

[30] Tajeri S (2022). *Theileria annulata* histone deacetylase 1 (TaHDAC1) initiates schizont to merozoite stage conversion. Sci. Rep..

[31] Jongejan F (1984). Cultivation of *Theileria*. I. Attempts to complete the cycle of *Theileria parva* in vitro. Vet. Q..

[32] Shaw MK (2003). Cell invasion by *Theileria* sporozoites. Trends Parasitol..

[33] Woods K, Perry C, Brühlmann F, Olias P (2021). *Theileria’s* strategies and effector mechanisms for host cell transformation: From invasion to immortalization. Front. Cell Dev. Biol..

[34] Shaw MK, Tilney LG (1995). The entry of *Theileria parva* merozoites into bovine erythrocytes occurs by a process similar to sporozoite invasion of lymphocytes. Parasitology.

[35] Paul AS, Egan ES, Duraisingh MT (2015). Host-parasite interactions that guide red blood cell invasion by malaria parasites. Curr. Opin. Hematol..

[36] Molina-Franky J, Patarroyo ME, Kalkum M, Patarroyo MA (2022). The Cellular and molecular interaction between erythrocytes and *Plasmodium falciparum* merozoites. Front. Cell. Infect. Microbiol..

[37] Elati K, Zweygarth E, Mhadhbi M, Darghouth MA, Nijhof AM (2022). Cultivation, cryopreservation and resuscitation of *Theileria annulata* transformed cells in serum-free media. Front. Vet. Sci..

[38] Shiels B (1992). Disruption of synchrony between parasite growth and host cell division is a determinant of differentiation to the merozoite in *Theileria annulata*. J. Cell Sci..

[39] Alvarez JA, Rojas C, Figueroa JV (2020). An overview of current knowledge on in vitro *Babesia* cultivation for production of live attenuated vaccines for bovine babesiosis in Mexico. Front. Vet. Sci..

[40] Zweygarth E, Josemans AI (2014). L-cysteine replaces microaerophilous culture conditions for the in vitro initiation of *Theileria equi*. Parasitol. Res..

[41] Lekishvili T, Campbell JJ (2018). Rapid comparative immunophenotyping of human mesenchymal stromal cells by a modified fluorescent cell barcoding flow cytometric assay. Cytometry A.

[42] Schindelin J (2012). Fiji: An open-source platform for biological-image analysis. Nat. Methods.

